# The Accuracy of Ultrasound Controlled Attenuation Parameter in Diagnosing Hepatic Fat Content

**DOI:** 10.2147/HMER.S411619

**Published:** 2023-06-09

**Authors:** Sebastiana Atzori, Yasmin Pasha, James B Maurice, Simon D Taylor-Robinson, Louise Campbell, Adrian K P Lim

**Affiliations:** 1 Department of Surgery and Cancer, Imperial College London, London, W1 1NY, UK; 2 Department of Medicine, Sassari University Hospital, Sassari, 07100, Italy; 3 UCL Institute for Liver and Digestive Health, Royal Free Hospital Campus, London, NW3 2QG, UK; 4 Office of the Clinical Director, Tawazun Health, London, W1G 9QN, UK; 5 Department of Imaging, Charing Cross Hospital, Imperial College Healthcare NHS Trust, London, W6 8RF, UK

**Keywords:** fibroscan, steatosis, diabetes, ballooning, liver, fibrosis

## Abstract

**Purpose:**

The Controlled Attenuation Parameter (CAP score) is based on ultrasonic properties of retropropagated radiofrequency signals acquired by Fibroscan^TM^ (Echosens, Paris, France). Since ultrasound propagation is influenced by the presence of fat, CAP score was developed to quantify steatosis. The aim of this study was to delineate the accuracy of CAP in diagnosing hepatic steatosis, compared to the gold standard of liver biopsy.

**Patients and Methods:**

A total of 150 patients underwent same-day liver biopsy and measurement of hepatic steatosis with Fibroscan. Only examinations with 10 satisfactory measurements, and an inter-quartile range of less than 30% of the median liver stiffness values were included for data analysis. Histological staging was then correlated with median values and Spearman correlation calculated. P values of <0.05 were considered statistically significant.

**Results:**

For diagnosis of hepatic steatosis (HS), CAP could predict the steatosis S2 with AUROC 0.815 (95% CI 0.741–0.889), sensitivity (0.81) and specificity (0.73) when the optimal cut-off value was set at 288 dB/m. CAP detected histological grade S3 with AUROC 0.735 (95% CI 0.618–0.851), sensitivity (0.71) and specificity (0.74), with a cut-off value of 330 dB/m. The AUROC for steatosis grade S1 was 0.741 (95% CI 0.650–0.824), with a cut-off value of 263 dB/m with sensitivity 0.75 and specificity 0.70. Univariate analysis showed a correlation between CAP and diabetes (p 0.048).

**Conclusion:**

The performance of CAP to diagnose steatosis severity decreases as steatosis progresses. CAP is associated with diabetes but not other clinical factors and parameters of the metabolic syndrome.

## Introduction

Metabolic-associated liver disease (MAFLD) is an increasing problem worldwide with a prevalence estimated to be 24%.^1^ Hepatic steatosis (HS) is present not only in MAFLD but also in chronic viral hepatitis, alcoholic liver disease and other aetiologies. Several studies demonstrated that HS plays an important role in fibrosis progression.^2^

Liver biopsy is the gold standard for the diagnosis and assessment of severity of HS, staging of fibrosis and is the only modality able to easily differentiate simple steatosis from steatohepatitis. It is an invasive and costly procedure and is prone to complications, some minor, such as pain, others more severe with a recorded risk of death of 0.01%.^3^

Therefore, several imaging, or laboratory-based methods have been developed to quantify HS non-invasively. Imaging modalities include ultrasound,^4^ computed tomography (CT) and magnetic resonance imaging (MRI).^5^,^6^ Recently, an additional ultrasound-based method, the Controlled Attenuation Parameter (CAP) has been developed to investigate HS non-invasively.^7^

CAP is included in the transient elastography system (TE), which uses vibration-induced elastic shear-waves for assessment of liver stiffness (Fibroscan^TM^, Echosens, Paris, France).^8^ For the 3.5 MHz TE M probe, the CAP algorithm calculates the attenuation of ultrasonic signals used for characterization of the shear-wave propagation. In contrast to conventional B-mode ultrasound, which is impaired by low sensitivity and difficulties in differentiating different grades of hepatic steatosis, CAP has shown repeatable performance for the detection and semi-quantification of steatosis in several biopsy-controlled clinical studies.^9^

A recent meta-analysis by Cao et al of 61 published studies suggested that CAP and TE could be used to screen for NAFLD in high-risk populations, although the cut-off values used for diagnosis may vary with body mass index and between populations.^10^

Despite this, liver biopsy remains the reference standard for non-invasive imaging techniques, although it is subject to sampling error and to variation with disease progression.^11^ This is particularly important where there is a fluctuating inflammatory component.^12^ To assess these issues and to provide further insight on the accuracy of CAP with respect to histology, liver biopsy should be performed as close as possible to the non-invasive measurements if these factors are to be minimised.^12^ Ideally therefore, comparative studies should have liver biopsy performed on the same day.

The aim of this study was to evaluate the CAP score in patients with liver disease who underwent same day liver biopsy and measurements with TE. Patients studied presented to our liver unit in consecutive order for liver biopsy without any prior selection. The importance of our study is the fact that CAP results were compared in all patients with the liver biopsy, the current gold standard.

## Materials and Methods

This was a prospective study approved by the Imperial College London Local Research Ethics Committee in accordance with the Helsinki Declaration on Human Rights of 1975 (REC reference 15/EE/0420).^13^ Written informed consent was obtained from all participants. Over a two-year study period, 160 consecutive patients with liver disease scheduled for a liver biopsy at the Liver Day-Care Unit of St. Mary’s Hospital, London, UK were investigated. Each of the patients underwent a hepatic US scan and were examined using Fibroscan^TM^ (Echosens, Paris, France) immediately before the liver biopsy. Ten subjects were excluded for technical reasons and therefore 150 were further studied.

### Transient Elastography

To assess liver stiffness measurements (LSM) as an indication of hepatic fibrosis, transient elastography was performed with Fibroscan^TM^ version 502 (Echosens, Paris, France) using a standard M probe. Examinations were performed by one of the three trained nurses with experience performing more than 5000 examinations. Only results with 10 satisfactory measurements, and an inter-quartile range of less than 30% of the median liver stiffness values were included for data analysis as valid. These parameters were considered necessary by Echosens for the accuracy of the measurements.^14^ CAP is a novel proprietary algorithm performed simultaneously with LSM using the ultrasonic signals acquired by the Fibroscan^TM^ to detect hepatic steatosis. CAP measures the ultrasound attenuation (go and return path) using signals acquired by the 3.5 MHz Fibroscan^TM^ probe, which measured the same liver area measured by LSM. The CAP values were expressed as dB/m and were considered valid if the LSM were valid.

### Morphological and Biological Parameters

For all patients, the following parameters were determined at the time of liver stiffness measurement. Clinical parameters included age, sex, body mass index (BMI), waist circumference, and clinical history. A blood sample was obtained one week prior the liver biopsy to quantify the platelet count, total bilirubin levels, gamma-glutamyl transpeptidase (GGT), aspartate aminotransferase (AST), alanine aminotransferase (ALT), alkaline phosphatase (ALP) and albumin.

The APRI score was calculated using the formula APRI =AST/AST ULN * 100/platelet count where AST ULN is the upper limit of the normal AST value (40 IU/L).^15^ The GGT-to-platelet ratio was also calculated.^16^

### Liver Histological Examinations

Histopathological staging for liver fibrosis served as the reference standard. Liver biopsies were fixed in formalin and embedded in paraffin. All the biopsies were read by one of two expert liver pathologists, who specialized in liver diseases, both with 30 years of experience. Liver fibrosis was evaluated semi-quantitatively in accordance with the METAVIR classification.^17^ Fibrosis was staged according to the NASH Clinical Research Network scoring system. The grade of steatosis was also defined according to Kleiner et al.^18^

### Statistical Analysis

Statistical analysis was performed using SPSS, Version 24.0 (IBM Statistics Chicago, IL) and MedCalc Software (MedCalc Software bvba, Ostend, Belgium). Quantitative variables were expressed as mean ± S.D or the median (IQR), and qualitative variables as absolute and relative frequencies. Baseline demographic, laboratory and biopsy characteristics of the patients are summarized using descriptive statistics. The distribution of the numerical variables was tested by Kruskal–Wallis nonparametric analysis of variance. The Central Limit Theorem was used to establish that normalized sum of independent variables tends toward a normal distribution. Bonferroni corrections were applied to normalise the data. Differences between numerical variables were analysed by parametric (*t*-test) or nonparametric tests (the Mann–Whitney or Kruskal–Wallis tests) according to the normality.

Multivariate analyses were used to assess the association of CAP with demographic, clinical and histological factors, and significant factors in the univariate analysis (P 0.01) were included in the multivariate analysis using multiple linear regressions. The diagnostic efficiency of CAP and LSM was analysed by computing the areas under the receiver operating characteristics curves (AUROC) and their 95% confidence interval (CI). The optimal cut-off value, sensitivity, specificity, positive predictive value/negative predictive value (PPV/NPV) were calculated by maximizing the Youden index (sensitivity, specificity – 1) to evaluate the diagnostic performance of CAP and LSM. Correlations between variables were calculated according to Spearman (regression coefficient r, r2).

## Results

### Patient Characteristics

Liver stiffness was evaluated by medians of TE in 160 subjects. Seven patients were excluded for inadequate histological specimen sample size (<1.5 cm and/or <6 portal tracts), one patient due to an unsuccessful LSM and two patients for both reasons. Data from 150 patients were analysed (86 males, 64 females; mean age, 49.76 years ±15.42). The main subjects’ characteristics are presented in Table 1. Eighty-nine patients had steatosis >5%. Steatosis grade was as follows: S0 (n = 61; 38%), S1 (n=41;27.3%), S2 (n = 42; 28%), and S3 (n = 7; 4.6%). Hepatocyte ballooning grade was 0 in n = 25 (16.6%), 1 in n = 34 (22.6%), 2 in n = 30 (20%). The median NAS score was 4 (Table 2) (3–5).

**Table 1:** Characteristics of the Study Cohort of 150 Patients

	Mean	Median	Minimum	Maximum
Sex, Female	64			
Age, years old	49.76 ±15.42	51.5	19	79
CAP score	278.13	277	52	400
BMI, kg/m^2^	27.88 ±5.56	27.56	16.61	47.04
Ethnicity				
White	88			
Black	17			
Asian	45			
Laboratory finding				
Platelet (103/mm^3^)	224.85±67.40	217.500	63	428
AST (IU/L)	71.40 ±42	99.11	9	817
ALT (IU/L)	90.73 ±61	115.54	7	960
Bilirubin (mg/dL)	16.21	11.4	5	101
GGT/Platelet	0.67 ±1.16	0.2660	0	8.22
Albumin (g/L)	38.43 ±6	39	23	74
INR	1.15 ±1.10	0.864	0.9	1.4
APRI	0.89 ±0.514	1.26	0.09	11.81
GGT (IU/L)	138.89 ±238.57	55.56	7	1588
Aetiology				
NAFLD	24			
NASH	40			
Chronic viral hepatitis B	26			
AIH	11			
DILI	12			
PBC	4			
PSC	4			
HIV with NAFLD	4			
HIV with NASH	4			
HIV with DILI	2			
HIV with abnormal LFTs	7			
Chronic viral hepatitis C	2			
OTHERS	10			
ALD	1			
FNH	1			
Wilson’s disease	1			
Haemochromatosis	2			
Obstetric cholestasis	1			
Sarcoidosis	1			
Sickle cell anemia	1			
Histiocytosis	1			
Cryptogenic	1			

**Note**: Data are mean ±standard deviation.

**Abbreviations**: AIH, autoimmune hepatitis; DILI, drug-induced liver injury; PBC, primary biliary cholangitis; PSC, primary sclerosing cholangitis; NAFLD, non-alcoholic fatty liver disease; LFTs, liver function tests; NASH, non-alcoholic steatohepatitis; ALD, alcohol liver disease; FNH, focal nodular hyperplasia, BMI, body max index; AST, aspartate aminotransferase; ALT; alanine aminotransferase; GGT, gamma-glutamyl transpeptidase.

**Table 2:** Characteristics of the Study Cohort

Total NAS Score	N
1	15
2	3
3	23
4	21
5	18
6	6
7	3
8	0
STEATOSIS	
0	60
1	41
2	42
3	7
BALLOONING	
0	25
1	34
2	30
LOBULAR INFLAMMATION	
0	27
1	88

### CAP for Assessment of Hepatic Steatosis, Ballooning and Lobular Inflammation

**Figure 1:**
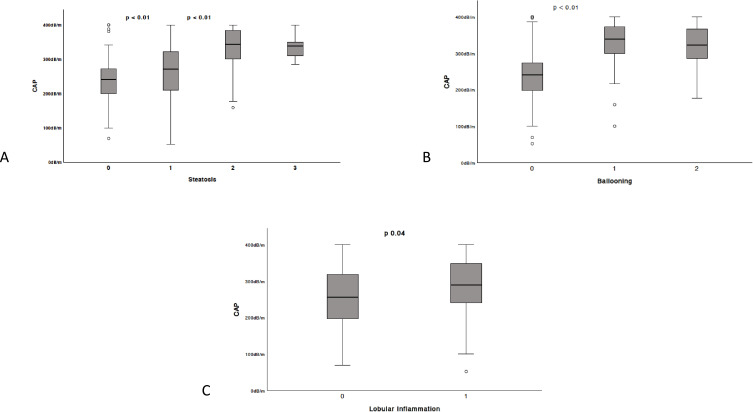
Boxplots of CAP measurements to detect (**A**) steatosis; (**B**) ballooning; (**C**) lobular inflammation for determination each grade. The boxes represent the interquartile range, and the thick lines within boxes, the median values measured using CAP. The error bars indicate the smallest and largest values within 1.5 box lengths of the 25th and 75th percentiles, respectively. The dots are outliers representing very large values that deviate significantly.

**Figure 2:**
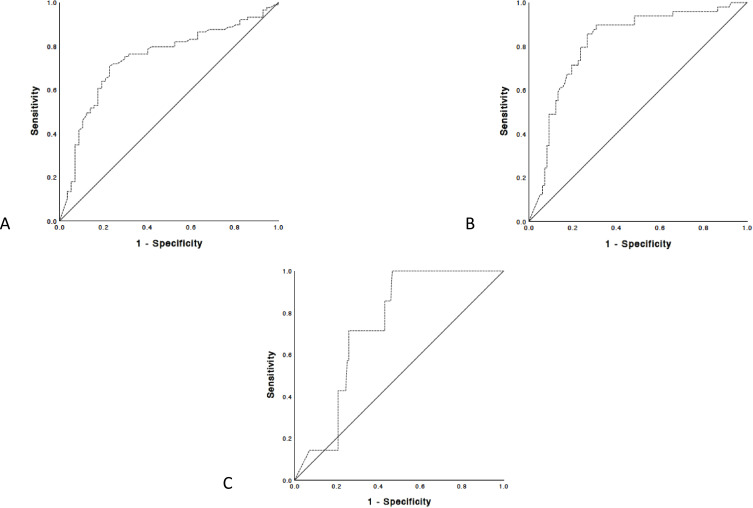
Receiver operating characteristic curves for the prediction of each steatosis grade. ROC curves for CAP for the diagnosis of (**A**) steatosis S1, (**B**) steatosis S2, (**C**) and steatosis S3.

The median range of CAP of the study population was 217.5 (100–400) dB/m. The median CAP values of stage of steatosis S1, S2 and S3 were 235 +-68.44 dB/m, 275 ± 81.92 dB/m, 332 ± 59.15 dB/m and 343 ± 39.26 dB/m, respectively (ANOVA: p = 0.01). The distributions of CAP for each degree of HS, ballooning and lobular inflammation are presented in Figures 1 and 2. The CAP values of S1 were significantly lower than those of S2 (p > 0.001), but the difference between S2 and S3 was not statistically significant (p = 0.07).

Spearman correlation between CAP score and histological results of liver biopsy demonstrated a correlation with steatosis (r^2^ 0.515, P = 0.001), ballooning (r^2^ 0.508, p = 0.001), NAS score (r^2^ 0.364, p = 0.01). There was a mild correlation with fibrosis (r^2^ 0.194, p = 0.01). There was no correlation with lobular inflammation (r^2^ 0.210, p = 0.06), or portal inflammation (r^2^-0.136, p = 0.10).

### Diagnostic Performance of CAP Score

For the diagnosis of HS, CAP could predict the steatosis ≥S2 with AUROC 0.815 (95% CI 0.741–0.889), sensitivity (0.81) and specificity (0.73) when the optimal cut-off value was set at 288 dB/m. CAP could detect the histological grade S3 with AUROC 0.735 (95% CI 0.618–0.851), sensitivity (0.71) and specificity (0.74) when the optimal cut-off value was set at 330 dB/m. The AUROC for a steatosis grade ≥S1 was 0.741 (95% CI 0.650–0.824), with a cut-off value set at 263 dB/m with sensitivity (0.75) and specificity (0.70) (Figure 2). Data are summarised in Table 3.

**Table 3:** Diagnostic Performance of CAP

Steatosis Grade	AUROC	Cut-Off	Sens	Spec
S1 n=102	0.741 0.650–0.824	263 dB/m	0.75	0.70
S2 n=43	0.815 0.741–0.889	288 dB/m	0.81	0.73
S3 n=7	0.735 0.618–0.851	330 dB/m	0.71	0.74

**Note**: Data are expressed with 95% confidence interval.

**Abbreviations**: AUROC, Area under the receiver operating characteristic; Sens, sensitivity; Spec, specificity; S1, steatosis grade 1; S2, steatosis grade 2; S3, steatosis, grade 3.

### Association of CAP with Demographic and Biochemical Factors

To observe the influence of demographic, biochemical values on CAP, we performed a univariate analysis that included the BMI, ALT, AST, APRI, GGT, GGT/platelet ratio, albumin, total bilirubin, platelets and IQR/Median ratio, IQR. The BMI (p 0.003), albumin (p 0.001) and platelets (p 0.001) showed a correlation with CAP score. However, only BMI significantly correlated with CAP in a subsequent multivariate analysis.

To observe the influence of histological data on CAP, we performed a univariate analysis that included fibrosis, steatosis, ballooning, portal inflammation, lobular inflammation. Steatosis (p 0.007), ballooning (p 0.009) showed a correlation with CAP. Data are summarised in Table 4.

**Table 4:** Univariate and Multivariate Analysis Showing Association of Clinical Parameters with CAP

Variables	CAP Univariate p	CAP Multivariate p
BMI	> 0.003 *	0.007*
Age	0.35	0.45
APRI (log^10^)	0.34	0.73
AST (log^10^)	0.20	0.67
ALT (log^10^)	0.16	0.24
Albumin	0.001*	0.56
Bilirubin (log^10^)	0.16	0.85
Platelets	0.001	0.87
GGT (log^10^)	0.66	0.95
GGT/Platelets (log^10^)	0.41	0.78
IQR/M	0.03 *	0.001*
Fibrosis score	>0.001 *	0.59
Steatosis	>0.007 *	0.0003*
Ballooning	0.009 *	0.004*
Lobular Inflammation	0.04	0.11
Portal Inflammation	0.72	0.33
NAS score	0.009 *	0.13

**Note**: *p <0.01.

**Abbreviations**: BMI, body max index; APRI, AST to Platelet ratio index; log10, logarithm10; AST, aspartate aminotransferase; ALT, alanine aminotransferase; GGT, gamma-glutamyl transpeptidase; IQR/M, interquartile range/median; NAS score, nonalcoholic fatty liver disease activity score.

To observe the influence of other pathologies on CAP, we performed a univariate analysis that included diabetes, hypercholesterolemia, and hypertension. Diabetes showed a correlation with CAP (p 0.048). There was no significant association between hypercholesterolemia (p 0.067) and hypertension (p 0.981). Data are summarised in Table 4.

### Discordance Between CAP and Histologic Steatosis Staging

For discordance analysis, three classes were defined: steatosis S1 < 260 dB/m, 260 to < 288 dB/m for the diagnosis of steatosis S2 and ≥ 288 dB/m for the diagnosis of S3 steatosis. Discordance of at least one grade between CAP and histology was observed in 59 (39%) patients. CAP predicted a higher steatosis grade in 6 cases and a lower steatosis grade in 53 cases.

The number of patients with discordance between no steatosis and steatosis S1 < 260 dB/m and histologic results was 21 (%), for S1 S2 between 264 and 330 was 13 (%), for S3 > 330 was 40 (%).

## Discussion

Non-alcoholic fatty liver disease (NAFLD) in a rising problem world-wide with a prevalence that is estimated to be 24%.^1^ HS is present not only in NAFLD but also in chronic viral hepatitis, alcoholic liver disease and other aetiologies. Several studies demonstrated that HS plays an important role in fibrosis progression.^2^

Liver biopsy is the gold standard for the diagnosis and assessment of severity of hepatic steatosis, staging of fibrosis and is the only modality able to differentiate bland steatosis from steatohepatitis. It is an invasive and costly procedure and is prone to complications, some minor, such as pain, others more severe with a recorded risk of death of 0.01%.

MRI exploits the difference of the resonance frequencies between water and fat. Several studies suggested that MRI-PDFF was sensitive in assessing changes in liver fat in the setting of a clinical trial. These data have been confirmed in multicentre studies in both adult and paediatric populations.^5^,^6^

Ultrasonography is the most used imaging method for the diagnosis of hepatic steatosis. Typical ultrasonography features are hyperechogenicity as compared to the right kidney parenchyma, distal attenuation, and the presence of areas of focal sparing.^4^ The degree of steatosis can be subjectively scored as absent, (score 0), mild (score 1), moderate (score 2), and severe (score 3).^19^ US can only detect steatosis with >2.5–20% liver fat content and its accuracy is reduced in fat patients and with kidney injury.^20^

According to the EASL guidelines,^21^ US is the preferred first-line diagnostic procedure for imaging of MAFLD, as it provides additional diagnostic information. Whenever imaging tools are not available or feasible (such as large epidemiological studies), serum biomarkers and scores are an acceptable alternative for the diagnosis of steatosis.

New ultrasound techniques are based on two properties of the US: attenuation and backscatter. Backscatter coefficient quantifies the scattered signal distribution based on backscattered signal. Attenuation of ultrasound signals is a gradual loss of signal strength due to absorption, reflection, refraction, scattering. Since ultrasound propagation is influenced by the presence of fat in the tissue, a new software has been developed to quantify steatosis. This parameter is based on the ultrasonic properties of the radiofrequency signals that are retropropagated and acquired by Fibroscan^TM^. This is the CAP score and is generated by a process based on vibration-controlled transient elastography (VCTE).^17^

In the present study, the clinical value of CAP for the detection of steatosis was evaluated in patients with liver disease and the results were compared with the histological results of the liver biopsies taken immediately after CAP measurements in the same session, using exactly the same intercostal approach for both techniques, something which has not been done before. This is important as HS is known to be a patchy condition and is known to affect the liver heterogeneously.^22^ This attempted to minimise the possible variation between the two different techniques. The optimal cut-off values of CAP for steatosis grades S1, S2 and S3 were 263 dB/m, 288 dB/m, 330 dB/m in the study which we performed.

The strength of our study is that it is the only one to correlate CAP scores with same day biopsy results, where in some studies correlation with MR-PDFF was used as the gold standard. Our study, however, is not the largest published one which used biopsy as a correlate. In the hallmark work, by Eddowes et al in 2019, the value of CAP for predicting fibrosis severity was assessed in a multicentre prospective study that included 450 patients with NAFLD evaluated by CAP/TE and liver biopsy, but not performed on the same day.^23^ The AUROCs of CAP to identify patients’ steatosis were as follows: for S ≥ S1-AUROC of 0.87; for S ≥ S2-0.77; while for S3 it was 0.70. Youden cut-off values were 302 dB/m for S ≥ S1, 331 dB/m for S ≥ S2, and 337 dB/m for S3. It is notable that the cut-off values varied a lot from our work that we present here and also from other published studies.^24^ An explanation could be the relatively small number of patients included in each study, the heterogeneity among groups regarding aetiology, overall steatosis prevalence, and also among steatosis severity groups.

In our study, AUROC values were 0.741, 0.815 and 0.735 respectively for steatosis S1, S2, and S3. Another meta-analysis included nine studies with 11 cohorts, totalling 1771 patients with CLD of diverse aetiologies.^9^ The summary sensitivity and specificity values were 0.78 and 0.79 for S ≥ 1; 0.85 and 0.79 for S ≥ 2; 0.83 and 0.79 for S3, respectively. The ROCs were 0.85 for S ≥ 1, 0.88 for S ≥ 2, and 0.87 for S3. The median optimal cut-off values of CAP for S ≥ 1, S ≥ 2, and S3 were 232.5 dB/m (range 214–289 dB/m), 255 dB/m (range 233–311 dB/m), and 290 dB/m (range 266–318 dB/m).

The performance of CAP for detecting steatosis (S≥ 1) is good, the AUROC usually being higher than 0.8. In populations with mixed aetiology of CLD, the AUROCs also remained high for diagnosing more severe steatosis (S2 and S3). However, in NAFLD population, the AUROCs for diagnosing moderate (S2) and severe (S3) steatosis decrease, sometimes being as low as 0.58, or even 0.37.^20^ In our study, CAP values of S1 were significantly higher and lower than those of S2 (p > 0.001), while the difference between S2 and S3 was not statistically significative (p = 0.07). This result is supported by previous studies.^25–28^

The suboptimal result of CAP in differentiating moderate and severe steatosis might be caused by the pathologists’ subjective interpretation between two consecutive stages and by the irregular distribution of steatosis in the liver. This has been an issue in most liver diseases and is particularly true in NAFLD.^24^

In our study, BMI, (p 0.003), albumin (p 0.001), platelets (p 0.001) showed a correlation with CAP score. However, only BMI is significantly correlated with CAP in a subsequent multivariate analysis. The presence of diabetes mellitus, but not hypercholesterolemia and hypertension, influence CAP values in the univariate analysis. These findings are in line with previous studies.^28^,^29^ Considering these findings, the authors^28^ propose an algorithm to correct the measured CAP values and to apply corrections including for diabetes mellitus, deducting 4.4 dB/m for each BMI unit over 25 kg/m^2^, or adding 4.4 dB/m for each BMI unit bellow 25 kg/m^2^.^30^

In our study, steatosis (p 0.007), ballooning (p 0.009), but, interestingly, not lobular or portal inflammation, showed a correlation with CAP.

Our results indicated that CAP score can provide useful information on the status of hepatic steatosis obtained by TE with or without inflammation. These findings could have clinical implications considering also the recently revised nomenclature of NAFLD in MAFLD. A recent study indicated that MAFLD individuals had a higher NAS than non-MAFLD individuals. Specifically, the difference in NAS originated from the severity of steatosis other than inflammation or ballooning degeneration. While the grades of inflammation and balloon degeneration were similar between the two groups, there was also no difference in the presence of NASH or significant fibrosis. According to the results of this study, metabolic dysfunction is associated with only steatosis, but no other histologic features in NAFLD. Thus, whether the renaming of NAFLD to MAFLD is rational still requires further studies on the dynamic histologic changes and long-term clinical outcomes between the MAFLD and non-MAFLD subgroups.

Given that meta-analyses have suggested that cut-off values for TE and CAP may vary with BMI, future studies should take this factor into account.^10^ Our study had too few patients to assess this statistically. Furthermore, recent studies have suggested that patterns of fat deposition might alter steatosis and progression of fibrosis between men and women.^31^ Further studies are required to assess this.

Our study had several limitations. First, the patients were from a liver centre. Therefore, the prevalence of liver steatosis could be higher than in the general population. Second, there may be a selection bias, where the indications for liver biopsy in our centre included patients with rare diseases. Third, precise correlation was lacking between the anatomical location of the US elasticity measurements and the segment of tissue that was obtained at biopsy for histological analysis, as not all patients had biopsies from the right lobe, where the LSM was done.

## Conclusion

The performance of CAP to diagnose steatosis severity decreases as the steatosis progresses. As observed in previous studies, CAP is associated with diabetes, but not with other clinical factors and parameters of the metabolic syndrome. Moreover, CAP results are influenced by the histological presence of steatosis and ballooning, but not by lobular or portal inflammation. This study adds further evidence in support of the clinical utility of CAP scores for non-invasive diagnosis of steatosis, and importantly with same day liver biopsy correlation.
